# Supplementary material for: Survivorship in Colorectal Cancer: A Cohort Study of the Patterns and Documented Content of Follow-Up Visits
Source: J Clin Med. 2020 Aug 24;9(9):2725. doi: 10.3390/jcm9092725 (PMC7563304; doi:10.3390/jcm9092725)
Supplement: Supplementary file 1 [file jcm-09-02725-s001.pdf]

Supplementary materials

Survivorship in Colorectal Cancer: A Cohort Study of the Patterns and Documented Content of Follow-Up Visits

Victoria Garwood [1,2], Karolina Lisy [1,3,4] and Michael Jefford [1,3,4,*]

1 Department of Cancer Experiences Research, Peter MacCallum Cancer Centre, Melbourne VIC 3000, AUS
2 Faculty of Medicine, Dentistry and Health Sciences, University of Melbourne, Parkville VIC 3010, AUS
3 Australian Cancer Survivorship Centre, Peter MacCallum Cancer Centre, Melbourne VIC 3000, AUS
4 Sir Peter MacCallum Department of Oncology, University of Melbourne, Parkville VIC 3010, AUS
* Correspondence: michael.jefford@petermac.org; Tel.: +61-3-8559-7902

Table S1. Audit Tool Assessing Elements of Survivorship Care, as outlined by the American Cancer Society [8].

Aspect of Survivorship Care	Record of Survivorship Care Element	Example of Appropriate Documentation
History and examination	1) New symptoms since last review	"I am pleased to report that X is currently asymptomatic."
	2) Physical examination of the abdomen, rectum, regional lymph nodes and/or another location	"Examination of the abdomen revealed a small incisional hernia…"
Post-treatment surveillance	3) Most recent results of colonoscopy	"X's most recent colonoscopy was on 1 January 2019 and was unremarkable."
	4) Most recent results of CEA testing	"A blood test taken this morning was reassuring, showing that X's CEA levels were within the normal range."
	5) Most recent results of CT imaging (chest, abdomen, pelvis) or equivalent	"CT imaging reported on 15 February 2019: NAD."
	6) No completed or suggested non-recommended tests without justification (e.g. PET/CT, MRI, other blood tests)	Appropriate documentation of PET/CT: "At X's last visit, a CT scan revealed a potential lesion in the liver. This was further characterised using PET/CT and was determined to be non-malignant."
Wider survivorship care	7) Discussion of primary cancer screening (i.e. breast, cervical and/or prostate cancer)	"Given that X is within the target screening group, I have strongly encouraged her to have a mammogram via BreastScreen Victoria."
	8) Discussion of physical changes (i.e. bowel, cognitive, dental/oral, fatigue, neuropathic, pain, reproductive, sexual and/or urinary)	"X reports opening their bowels approximately three times a day, which is normal for them."
	9) Discussion of psychological changes (i.e. anxiety, body image, depression, distress, fear of recurrence and/or guilt)	"X appears concerned about the likelihood of recurrence and it has kept them up at night recently."
	10) Discussion of social and/or practical changes (i.e. employment, finances,	"X is coping well with their return to full-time employment, which is very encouraging."

	intimacy, quality of life, relationships and/or spirituality)	
	11) Discussion of regular health checks and/or screening for other non-communicable diseases (e.g. cardiovascular disease and/or osteoporosis)	"I have spoken to X about living well after cancer, in particular the importance of regularly attending the GP and having regular screening as appropriate for her gender and age."
	12) Discussion of lifestyle behaviours (i.e. body weight, nutrition and diet, physical activity, smoking, alcohol intake and/or UV radiation)	"I have provided X with a booklet from Cancer Council Australia about living well after cancer through healthy eating and exercise."
Care coordination	13) Letter to PCP and/or another specialist outlining outcome of follow-up visit	N/A
	14) Designation of roles and/or responsibilities to others involved in care (e.g. PCP to action, specialist to action)	"X has expressed to me that they would like to have their imaging and blood tests done closer to home. Would you be able to facilitate this?"

Abbreviations. CEA, carcinoembryonic antigen; CT, computed tomography; e.g., for example; i.e.; in other words; MRI, magnetic resonance imaging; N/A, not applicable; NAD, no abnormality detected; PCP, primary care physician; PET, positron electron tomography; UV, ultraviolet.

Table S2. Frequency of Follow-Up Visits in the Study Population (n = 48) in the First Year (360 days), Stratified by Treatment.

Frequency of visits	Surgery only n (%)	Surgery + receipt of neoadjuvant and/or adjuvant therapy n (%)	Overall n (%)
1	1 (5%)	1 (4%)	2 (4%)
2	2 (10%)	2 (7%)	4 (8%)
3	9 (43%)	6 (22%)	15 (31%)
4	6 (29%)	9 (33%)	15 (31%)
5	3 (14%)	4 (15%)	7 (15%)
6	0	0	0
7	0	3 (11%)	3 (6%)
8	0	2 (7%)	2 (4%)
Total	21	27	48

Table S3. Documented Content of Follow-Up Visits in the Study Population (n = 48) in the First Year (360 days), Stratified by Treatment.

Aspect of Survivorship Care Element (see Table A1)	Surgery only n (%)	Surgery + receipt of neoadjuvant and/or adjuvant therapy n (%)	Overall n (%)
1) New symptoms since last review	19 (90%)	27 (100%)	46 (96%)
2) Physical examination	12 (57%)	18 (67%)	30 (63%)
3) Colonoscopy results	9 (43%)	18 (67%)	27 (56%)
4) CEA results	17 (81%)	26 (96%)	43 (90%)
5) CT results	15 (71%)	22 (81%)	37 (77%)

6) Additional investigations appropriately justified	21 (100%)	22 (81%)	43 (90%)
7) Discussion of screening for other primary cancers	0	0	0
8) Discussion of physical changes	17 (81%)	24 (89%)	41 (85%)
9) Discussion of psychological changes	0	4 (15%)	4 (8%)
10) Discussion of social and/or practical changes	3 (14%)	12 (44%)	15 (31%)
11) Discussion of regular health checks and/or screening	0	0	0
12) Discussion of lifestyle behaviours	9 (43%)	13 (48%)	22 (46%)
13) Letter to PCP and/or another specialist	21 (100%)	27 (100%)	48 (100%)
14) Designation of roles and/or responsibilities to others	3 (14%)	12 (44%)	15 (31%)

Abbreviations. CEA, carcinoembryonic antigen; CT, computed tomography; PCP, primary care physician.
